# Bronchoscopy-associated dissemination of pulmonary nocardiosis caused by *Nocardia terpenica* in an immunocompetent patient with bronchiectasis: A case report

**DOI:** 10.1097/MD.0000000000045875

**Published:** 2025-11-07

**Authors:** Jie Tian, Jingjun Dong, Gaobing Yu, Wei Guan

**Affiliations:** a Department of Respiratory Medicine, The Central Hospital of Baoan, Shenzhen, Guangdong Province, China; b Department of Clinical Microbiology, The Central Hospital of Baoan, Shenzhen, Guangdong Province, China.

**Keywords:** bronchoscopy complication, immunocompetent host, *Nocardia terpenica*, pulmonary nocardiosis, therapeutic duration

## Abstract

**Rationale:**

Bronchoscopy with bronchoalveolar lavage (BAL) is essential for diagnosing pulmonary infections; however, its potential to iatrogenically disseminate a localized Nocardia infection represents a severe and unreported risk. This case aims to alert clinicians to this danger and underscore essential therapeutic lessons for disseminated disease.

**Patient concerns:**

A 51-year-old immunocompetent woman with longstanding bronchiectasis presented with 1-day of hemoptysis and a 40-year history of chronic cough and sputum production. These symptoms had worsened over the preceding 2 months despite broad-spectrum antibiotic therapy.

**Diagnoses:**

Bronchiectasis was diagnosed based on chronic respiratory symptoms and characteristic computed tomography findings. *Nocardia terpenica* infection was confirmed by BAL fluid culture and metagenomic next-generation sequencing. Within 24 hours post-BAL, the patient developed fever, respiratory failure, and new bilateral consolidations on computed tomography, indicating procedure-related disseminated nocardiosis.

**Interventions:**

Diagnostic bronchoscopy with BAL was performed. Therapeutically, the patient received a total of 24 days of intensive combination therapy with intravenous imipenem/cilastatin and oral trimethoprim–sulfamethoxazole (TMP–SMX), followed by sequential long-term oral TMP–SMX monotherapy.

**Outcomes:**

The initial 10-day course of combination therapy led to rapid clinical improvement, with resolution of fever and respiratory failure within 3 days, and normalization of C-reactive protein levels by day 10. Radiographic improvement was also evident. However, relapse (recurrent fever and malaise) occurred promptly within 3 days after de-escalation to TMP–SMX monotherapy. After reinstitution of imipenem/cilastatin plus TMP–SMX for an additional 14 days (totaling 24 days of intensive therapy), the patient achieved sustained clinical and radiographic remission. She was successfully discharged on long-term TMP–SMX monotherapy and remained well at the 2-month follow-up.

**Lessons:**

This is the first report suggesting that bronchoscopy, particularly BAL, can disseminate a localized airway Nocardia infection, causing acute disseminated pulmonary nocardiosis. Extreme caution is warranted when performing bronchoscopy in bronchiectasis patients with suspected or confirmed nocardiosis. For disseminated pulmonary nocardiosis, intensive combination therapy for at least 3 weeks is mandatory to prevent relapse, regardless of a rapid initial response.

## 1. Introduction

Pulmonary nocardiosis is an uncommon but potentially life-threatening bacterial infection caused by aerobic actinomycetes of the genus Nocardia. It classically affects immunocompromised individuals, particularly those with impaired cell-mediated immunity (e.g., transplant recipients, human immunodeficiency virus (HIV)/acquired immunodeficiency syndrome, chronic corticosteroid use).^[1]^ Infection in immunocompetent hosts is relatively uncommon, accounting for roughly 15% to 30% of cases, often associated with underlying structural lung disease like chronic obstructive pulmonary disease or, less frequently, bronchiectasis.^[2]^ *Nocardia terpenica* is an environmental species rarely implicated in human disease; reports of pulmonary infection are exceptionally scarce.^[3]^ Diagnosis is challenging due to nonspecific clinical and radiological presentations, which can mimic tuberculosis, fungal infections, or malignancy, often including nodules, masses, cavitation, or consolidation.^[4]^ Bronchoscopy with bronchoalveolar lavage (BAL) is a key diagnostic procedure, facilitating microbiological sampling.^[5]^ Standard treatment involves prolonged (months) sulfonamide-based therapy, often initiated with a carbapenem (like imipenem) or Linezolid for severe or disseminated disease^[6]^ We present a highly unusual case of pulmonary nocardiosis caused by *N terpenica* in an immunocompetent patient with localized bronchiectasis, complicated by apparent bronchoscopy-induced dissemination and illustrating critical lessons regarding therapeutic duration.

## 2. Case presentation

A 51-year-old woman presented to our hospital with a 1-day history of hemoptysis (approximately 50 mL of fresh blood). She reported a 40-year history of chronic cough productive of white sputum. Two months prior to admission, her cough and sputum production worsened. She sought treatment at local clinics and a county hospital, receiving multiple courses of antibiotics (including piperacillin/tazobactam and ceftazidime) targeting presumed bronchiectasis exacerbation, without improvement. She denied fever, night sweats, significant weight loss, dyspnea, or chest pain prior to the hemoptysis. Past medical history was significant only for chronic bronchiectasis, diagnosed years earlier based on symptoms and imaging. She had no history of diabetes, HIV infection, malignancy, chronic steroid use, or other known immunodeficiencies.

**Figure 1.**
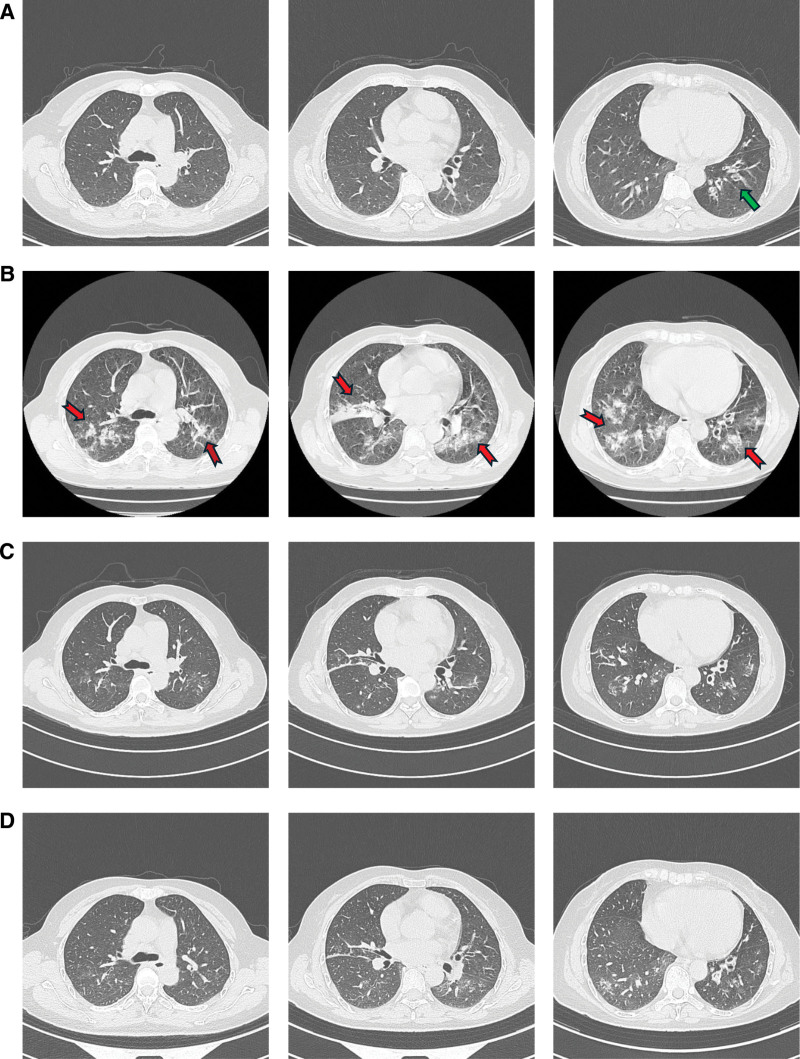
Serial chest CT imaging. (A) Admission (day 1): localized bronchiectasis in left lower lobe (green arrow); (B) 36 hours post-bronchoscopy (day 9): new bilateral consolidations distributed predominantly along bronchovascular bundles (red swallow-tailed arrows); (C) day 18: marked resolution after initial therapy; (D) day 38: continued improvement after extended combination therapy. CT = computed tomography.

On admission, she was afebrile (36.8°C), hemodynamically stable, and alert. Respiratory examination revealed coarse crackles over the left lower lung zone. Initial laboratory investigations showed a white blood cell count of 6.73 × 10⁹/L (reference 4.0–10.0), hemoglobin 127 g/L (110–150), and platelet count 330 × 10⁹/L (100–350), with lymphocyte subsets showing CD3+ T cells at 1604 cells/μL (955–2860), CD4+ T cells at 1050 cells/μL (550–1440), and CD8+ T cells at 542 cells/μL (320–1250). Immunoglobulin levels were normal (IgG 13.11 g/L [7–16], IgA 2.88 g/L [0.7–4.0], IgM 0.96 g/L [0.4–2.3]), as were complement components (C3 1.08 g/L [0.9–1.8], C4 0.17 g/L [0.1–0.4]). Inflammatory markers showed C-reactive protein at 0.80 mg/L (0–6.0) and procalcitonin < 0.01 ng/mL (0–0.05). Infection screening was negative for HIV (0.15 S/CO; <1.0), hepatitis B (HBsAg 0.01 IU/mL; <0.05), hepatitis C (0.02 S/CO; <1.0), and syphilis (0.06 S/CO; <1.0), while autoimmunity testing showed negative antinuclear antibody, negative anti-ds-DNA, and rheumatoid factor < 10.0 kU/L (0–30.0), collectively confirming immunocompetence without evidence of systemic inflammation or immunosuppressive conditions (Table 1). Chest computed tomography (CT) showed localized cylindrical bronchiectasis involving the left lower lobe, without nodules, masses, cavitation, or consolidation elsewhere (Fig. 1A).

**Table 1:** Admission laboratory profile: hematology, immunology, inflammation, infection, and autoimmunity screening.

Laboratory test	Result	Unit	Reference range	Status
Complete blood count				
WBC	6.73	×10⁹/L	4.0–10.0	Normal
HGB	127	g/L	110–150	Normal
PLT	330	×10⁹/L	100–350	Normal
Neutrophils	3.58	×10⁹/L	1.8–7.2	Normal
Lymphocytes	2.39	×10⁹/L	0.8–4.0	Normal
Eosinophils	0.22	×10⁹/L	0.02–0.50	Normal
Lymphocyte subsets				
CD45+CD3+ T cells	1604	cells/μL	955–2860	Normal
CD3+CD4+ T cells	1050	cells/μL	550–1440	Normal
CD3+CD8+ T cells	542	cells/μL	320–1250	Normal
CD4+/CD8+ ratio	1.94	ratio	0.71–2.78	Normal
CD19+ B cells	217	cells/μL	100–500	Normal
CD16 + 56+ NK cells	385	cells/μL	100–500	Normal
Immunoglobulins				
IgG	13.11	g/L	7–16	Normal
IgA	2.88	g/L	0.7–4.0	Normal
IgM	0.96	g/L	0.4–2.3	Normal
Complement system				
C3	1.08	g/L	0.9–1.8	Normal
C4	0.17	g/L	0.1–0.4	Normal
Inflammatory markers				
CRP	0.80	mg/L	0–6.0	Normal
PCT	<0.01	ng/mL	0–0.05	Normal
Infection screening				
HIV Ab	0.15	S/CO	<1.0	Negative
HBsAg	0.01	IU/mL	<0.05	Negative
Anti-HCV	0.02	S/CO	<1.0	Negative
Syphilis (TP-CLIA)	0.06	S/CO	<1.0	Negative
Autoimmunity screening				
ANA	Negative	–	Negative	Normal
Anti-ds-DNA	Negative	–	Negative	Normal
RF	<10.0	kU/L	0–30.0	Normal

ANA = antinuclear antibody, anti-HCV = hepatitis C virus antibody, CRP = C-reactive protein, HBsAg = hepatitis B surface antigen, HGB = hemoglobin, HIV Ab = human immunodeficiency virus antibody, IgA = immunoglobulin A, IgG = immunoglobulin G, IgM = immunoglobulin M, PCT = procalcitonin, PLT = platelets, RF = rheumatoid factor, TP-CLIA = *Treponema pallidum* chemiluminescence immunoassay, WBC = white blood cell.

**Figure 2.**
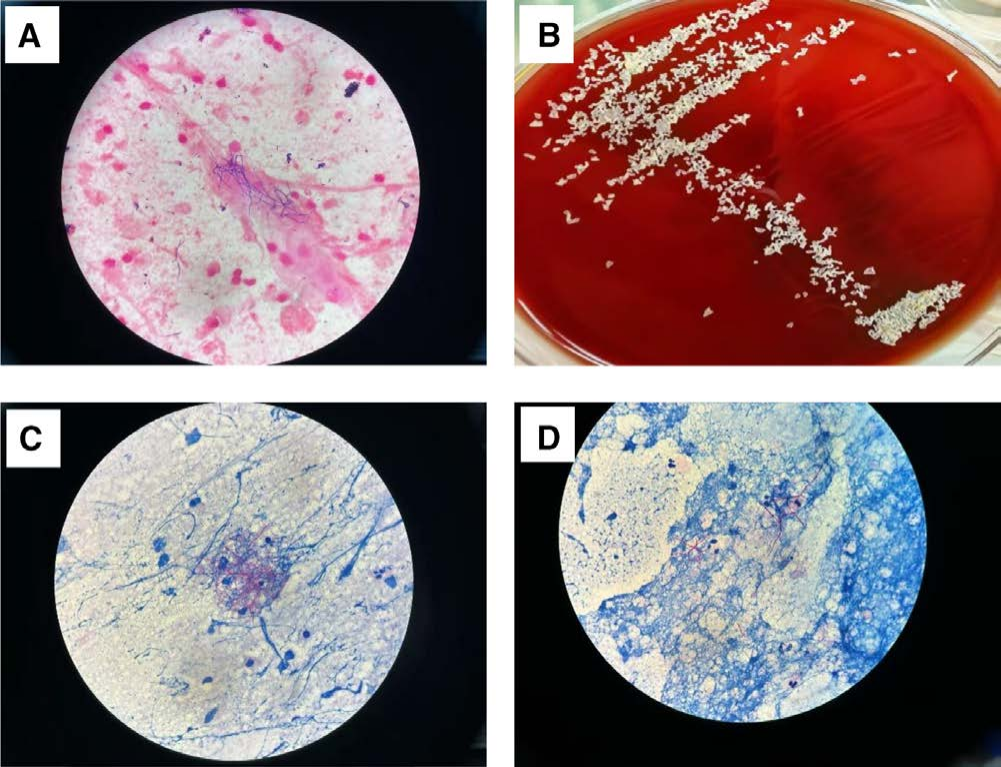
Microbiological identification of *Nocardia terpenica*. (A) Gram stain demonstrating gram-positive, beaded, branching filaments with characteristic irregular staining and fragmentation, consistent with Nocardia morphology (1000×, oil immersion). (B) Macroscopic colony characteristics on sheep blood agar after 72-hour incubation: dry, chalk-white colonies with irregular cerebriform margins and adherent growth. The reverse side exhibits distinct orange-yellow pigmentation. (C, D) Weakly acid-fast stains (modified Kinyoun method) showing slender, 90°-angled branching acid-fast bacilli (red) against blue background, without terminal bulbous structures (C) and the beaded filament structure in (D; 1000×, oil immersion). Technical specifications: Gram stain: crystal violet-safranin method; acid-fast stain: 1% sulfuric acid decolorization; culture: 5% sheep blood agar, 35°C, ambient air.

**Figure 3.**
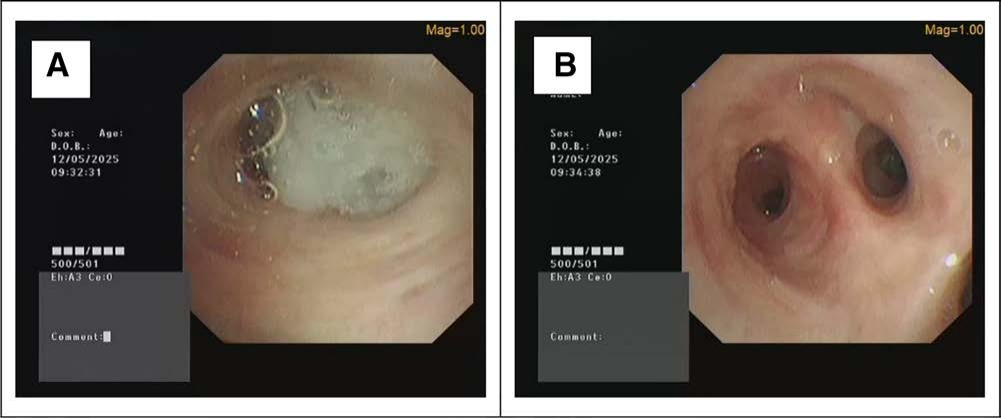
Bronchoscopic findings in left lower lobe bronchus. (A) Initial view showing copious purulent secretions completely occluding the bronchial lumen. (B) Post-suction view revealing diffuse mucosal erythema without ulceration, exudate, or active bleeding, consistent with chronic inflammatory changes.

She was admitted with a diagnosis of bronchiectasis exacerbation with hemoptysis. Considering the structural lung disease and common pathogens (especially *Pseudomonas aeruginosa*), empirical intravenous (IV) levofloxacin (500 mg daily) was initiated. Upon admission, deep expectorated sputum samples were obtained for routine bacterial culture and microscopic examination. On hospital day 3, the clinical microbiology laboratory telephoned a critical preliminary finding: microscopic examination revealed abundant branching filamentous bacilli in the sputum smear (Fig. 2), though definitive identification was pending. Based on this finding, bronchoscopy with BAL was recommended to clarify the pathogen identity and assess airway conditions. However, the patient initially declined bronchoscopy due to apprehension about procedural risks. Consequently, a second deep sputum sample was collected as an alternative diagnostic approach and submitted for metagenomic next-generation sequencing (mNGS). The mNGS analysis identified *Nocardia* spp. with 11,276 sequence reads, providing crucial preliminary pathogen identification. Following detailed counseling with the patient and family members, informed consent for bronchoscopy was provided (Written informed consent was obtained from the patient for both the bronchoscopic procedure). To evaluate the source of hemoptysis and airway conditions, electronic bronchoscopy was performed. This revealed copious purulent secretions occluding the left lower lobe bronchi, particularly the basal segments (Fig. 3). Bronchial washing, BAL from the left lower lobe basal segment, and bronchial brushing were performed. BALF was sent for mNGS, culture, and cytology. The brush smear cytology showed branching filamentous bacteria suggestive of *Nocardia* spp. BALF mNGS (3,01,951 sequence reads) and subsequent culture confirmed *N terpenica*, with matrix-assisted laser desorption/ionization time-of-flight mass spectrometry providing definitive species identification.

Remarkably, within 24 hours of the bronchoscopy procedure, the patient developed high fever (39.5°C), significant malaise, anorexia, increased cough productive of purulent yellow sputum, chest tightness, shortness of breath, and respiratory failure. Repeat laboratory tests showed significant leukocytosis (WBC 23.61 × 10⁹/L), elevated CRP (120.05 mg/L) and PCT (0.12 ng/mL), type I respiratory failure (partial pressure of oxygen, arterial (PaO_2_) 55 mmHg, partial pressure of carbon dioxide, arterial (PaCO_2_) 41 mm Hg and oxygenation index 261 under non-oxygen inhalation conditions). Urgent repeat chest CT (approximately 36 hours post-bronchoscopy) revealed dramatic new multifocal, patchy consolidations and ground-glass opacities distributed along the bronchovascular bundles in *both* lungs, consistent with disseminated pulmonary infection (Fig. 1B). This represented a stark contrast to the pre-bronchoscopy scan, showing only localized bronchiectasis.

A diagnosis of disseminated pulmonary nocardiosis, likely precipitated by the bronchoscopic procedure causing iatrogenic spread of the pathogen, was made. Guided by the Sanford Guide to Antimicrobial Therapy,^[6]^ treatment was immediately started with IV imipenem/cilastatin (1 g every 8 hours) plus high-dose oral trimethoprim–sulfamethoxazole (TMP 15 mg/kg/d SMX 75 mg/kg/d divided every 8 hours). A rapid clinical response ensued. Fever resolved by day 3 of therapy. Symptoms (cough, sputum, malaise, shortness of breath) improved significantly. Inflammatory markers normalized by day 10 of treatment with imipenem/cilastatin and TMP–SMX (WBC 7.01 × 10⁹/L, CRP 5.79 mg/L). A CT scan on day 10 of treatment (day 18 from admission) showed marked resolution of the disseminated consolidations (Fig. 1C). Feeling clinically well and with resolving radiology, the patient was discharged on day 10 of combination therapy to complete a planned prolonged course with oral TMP–SMX monotherapy (same dose).

Unexpectedly, the patient presented again to the emergency department on the *third day* after discharge (day 21 overall from admission) with recurrent fever (38.8°C) and malaise. She was readmitted. Key considerations were treatment failure due to resistance or inadequate drug levels/duration. The *N terpenica* isolate recovered earlier underwent formal antibiotic susceptibility testing (broth microdilution). Results confirmed susceptibility to both imipenem (minimum inhibitory concentration [MIC] ≤ 2 µg/mL) and TMP–SMX (MIC ≤ 0.5/9.5 µg/mL). Serum trough levels of sulfamethoxazole were measured and found to be within the therapeutic range (target 100–150 µg/mL). This excluded primary resistance and subtherapeutic TMP–SMX levels as causes of relapse.

**Figure 4.**
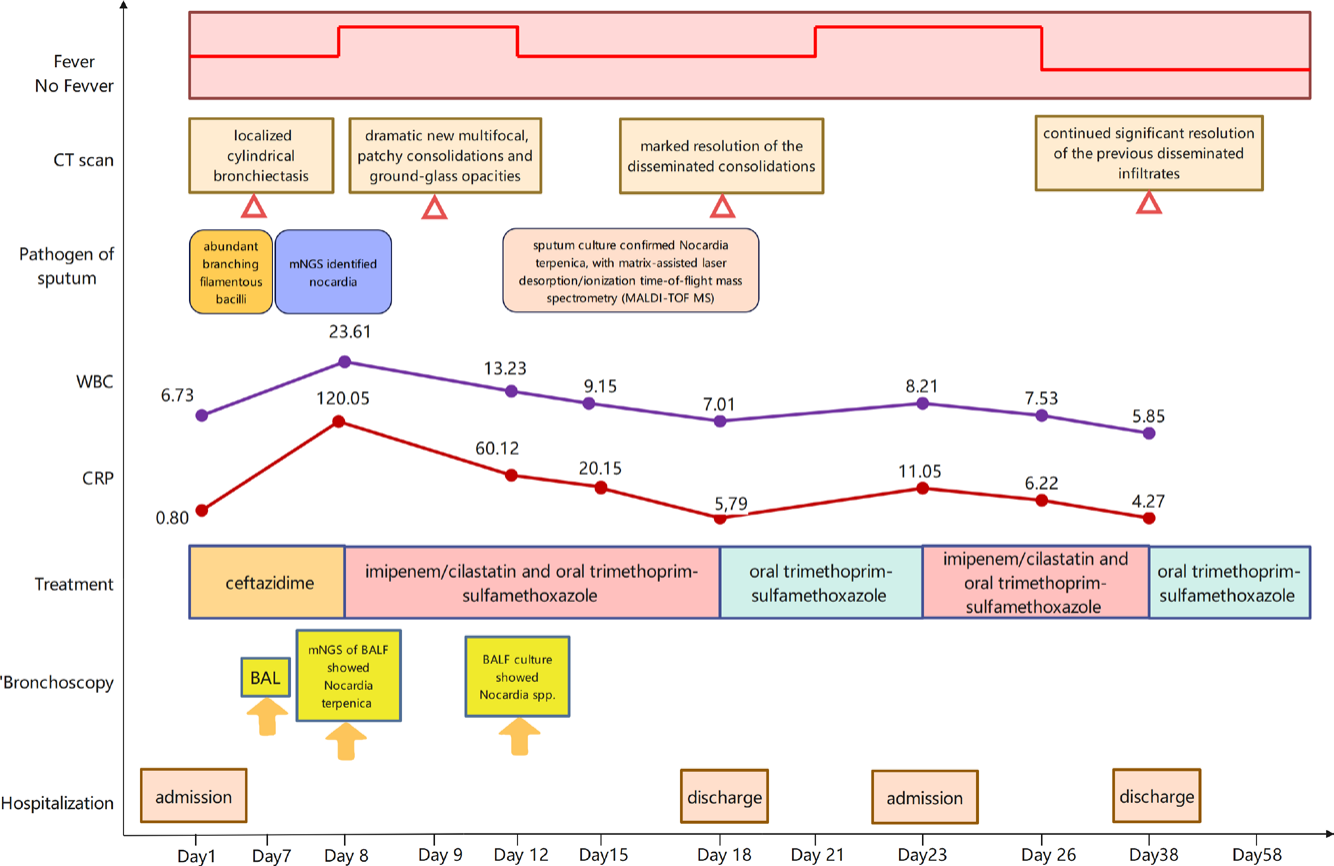
Clinical course and management timeline of the patient. The diagram illustrates key clinical events, interventions, and outcomes from admission to follow-up. CRP = C-reactive protein, CT = computed tomography, WBC = white blood cell.

Consequently, the relapse was attributed to an insufficient duration of the *intensive combination phase* for disseminated disease. The initial 10-day course, though effective acutely, was inadequate for sterilization. The combination regimen of IV imipenem/cilastatin plus oral TMP–SMX was restarted. The patient responded rapidly again, with fever resolving within 72 hours. She received a *total* of more than 3 weeks (24 days) of this intensive combination therapy (10 days initial admission + 14 days readmission). At the end of this period, she was afebrile, asymptomatic, and her inflammatory markers (WBC, CRP) were normal. A follow-up CT scan showed continued significant resolution of the previous disseminated infiltrates (Fig. 1D). She was discharged on high-dose oral TMP–SMX monotherapy (TMP 15 mg/kg/d) for planned long-term suppression (minimum 6 months total therapy). At the 2-month outpatient follow-up post-discharge, she remained clinically well, afebrile, and without recurrence of symptoms (Fig. 4).

## 3. Discussion

Pulmonary nocardiosis remains a relatively uncommon infection, classically associated with compromised host defenses, particularly impaired cell-mediated immunity in conditions such as solid organ transplantation, hematological malignancies, HIV/acquired immunodeficiency syndrome, and chronic corticosteroid therapy.^[1,2]^ Notably, our patient starkly contrasts this typical profile. Comprehensive admission immunological evaluations – including lymphocyte subset analysis (normal CD3+, CD4+, and CD8+ counts), quantitative immunoglobulins (normal IgG, IgA, IgM), complement levels (normal C3, C4), and negative serology for HIV, hepatitis, syphilis, and autoimmunity – confirmed her immunocompetent status. This places her among the minority (approximately 15–30% of pulmonary nocardiosis cases) occurring without overt immunodeficiency.^[1,2]^ The rarity is further compounded by the causative organism: while Nocardia asteroides and *N brasiliensis* account for most human nocardial infections,^[2,7]^ our case involved *N terpenica*, an environmental species first described in 2007.^[8]^ Clinical reports of *N terpenica* are exceptionally scarce, predominantly documenting extrapulmonary manifestations such as catheter-related bloodstream infections or central nervous system involvement^[3]^ Pulmonary infections caused by *N terpenica* are exceptionally scarce in the medical literature, To our knowledge, only 1 other immunocompetent case of pulmonary nocardiosis attributed to *N terpenica* has been reported – a milder presentation previously reported by our group.^[9]^ In contrast, this case demonstrates dramatically more severe disease characterized by post-procedural dissemination and a complex treatment course requiring extended intensive therapy, highlighting the unpredictable pathogenicity of this rare organism.

Beyond microbiological rarity, this case underscores the critical importance of identifying specific risk factors for nocardiosis in immunocompetent individuals. Chronic structural lung disease, particularly bronchiectasis, as present here, is a well-documented predisposition.^[10]^ The distorted airways, impaired mucociliary clearance, and persistent microbial colonization inherent in bronchiectasis create a permissive environment for opportunistic pathogens like Nocardia. Consequently, when bronchiectasis patients show suboptimal response to extended broad-spectrum antibiotics targeting common pathogens (e.g., *P aeruginosa*), diagnostic considerations should expand to include mycobacterial infections (tuberculosis and nontuberculous mycobacteria), fungal pathogens (e.g., Aspergillus), and notably nocardiosis, particularly when standard evaluations for tuberculosis and fungi are negative. Furthermore, environmental exposure history is essential. Our patient’s occupation as a farmer involved frequent soil exposure – the primary reservoir of Nocardia species.^[11]^ This represents a significant yet underappreciated risk factor, emphasizing the need for detailed environmental histories in refractory respiratory infections, even without classic immunosuppression.

Bronchoscopy with BAL remains an essential diagnostic tool for pulmonary infections, particularly for identifying uncommon pathogens like Nocardia.^[12]^ Although localized pathogen spread within the lavaged segment is a recognized complication, dissemination to distant or contralateral lobes is exceptionally rare. The dissemination pattern observed here is highly unusual: within 24 hours of BAL confined to the left lower lobe basal segment, the patient developed systemic symptoms and new multifocal consolidations involving both the ipsilateral left upper lobe and contralateral right upper/lower lobes. This extensive spread beyond the lavaged segment is unprecedented in both our experience and the literature. Several factors argue against procedural error: highly experienced bronchoscopists; strict adherence to protocols including initial contralateral airway inspection; BALF volume (80 mL) within guideline recommendations (60–120 mL); routine bronchoscopy in tuberculosis patients (a pathogen with known dissemination propensity) without comparable spread; and crucially, our prior BAL procedure in an immunocompetent *N terpenica* case caused no dissemination.^[9]^

To our knowledge, this constitutes the first documented case of bronchoscopy-induced widespread pulmonary nocardiosis dissemination. The pathogenesis of this dramatic dissemination likely arises from a unique confluence of patient-specific factors and extraordinary pathogen burden. Crucially, comparative metagenomic sequencing revealed a massive surge in bacterial load following BAL: pre-procedural deep sputum mNGS detected significant *N terpenica* burden (11,276 sequence reads), while post-procedural BALF mNGS showed an explosive increase (3,01,951 sequence reads). This >26-fold escalation demonstrates how the BAL procedure triggered sudden, massive pathogen release into the airways. Although systemic immunity had contained the infection regionally, bronchoscopy served as the catalyst – pressurized instillation and repeated aspiration in this heavily colonized segment forcibly dislodged organisms. This effect was amplified by underlying bronchiectasis – characterized by structural airway damage, compromised local immunity, and impaired mucociliary clearance – creating ideal conditions for bronchogenic spread. The bilateral upper lobe involvement remains particularly notable, defying expected gravity-dependent fluid distribution. We speculate that vigorous suctioning during BAL generated Nocardia-laden aerosols, facilitated by mycolic acids in the bacterial cell wall. These long-chain α-alkyl, β-hydroxylated fatty acids constitute the primary hydrophobic components of the outer membrane.^[13]^ By forming a hydrophobic barrier, mycolic acids not only confer environmental persistence but also promote aerosol formation and stability.^[14]^ During BAL, repeated suction likely produced abundant pathogen-containing aerosols.^[15]^ The hydrophobic nature of mycolic acids enables prolonged suspension of these aerosols in air, ultimately facilitating the suspension and airborne transmission of organisms during the procedure, explaining disseminated lesions in bilateral upper lobe areas, which are more likely to receive and retain aerosolized particles.

This case reveals a critical, previously unrecognized hazard: BAL in bronchiectasis patients harboring high-load *Nocardia* airway colonization carries a substantial risk of precipitating severe disseminated pulmonary nocardiosis, even with technically flawless execution. Heightened caution is therefore warranted when considering bronchoscopy, particularly BAL, in such patients. When BAL remains essential for diagnosis despite noninvasive tests (e.g., sputum mNGS/culture) strongly indicating *Nocardia*, implement aggressive risk mitigation: minimize lavage volume (e.g., 60 mL at lower guideline range), avoid vigorous suctioning in grossly purulent areas, limit suction cycles to reduce aerosol generation, and ensure optimal post-procedure drainage through positioning and coughing. For patients with confirmed or suspected Nocardia infection undergoing BAL, clinicians must proactively address aerosol dissemination risks through these measures to prevent iatrogenic spread.

Regarding treatment, current guidelines show important convergences in initial antimicrobial selection for Nocardia infection. Both major sources recommend combination therapy for severe or disseminated infection. The American Society of Transplantation guidelines for solid organ transplant recipients endorse TMP–SMX monotherapy for mild to moderate pulmonary infection, reserving combination regimens (imipenem/cilastatin plus amikacin or TMP–SMX) for severe disease, central nervous system involvement, or disseminated infection.^[16]^ Similarly, the Sanford Guide recommends initial TMP–SMX plus imipenem/cilastatin for severe or disseminated pulmonary nocardiosis.^[17]^ Our patient presented with acute severe disseminated disease (high fever, respiratory failure, multifocal consolidations) post-BAL. We empirically initiated the Sanford-recommended regimen of IV imipenem/cilastatin plus high-dose oral TMP–SMX pending susceptibility. Response was remarkably rapid: fever resolved within 72 hours, inflammatory markers normalized, and CT showed significant resolution of disseminated infiltrates by day 10 of combination treatment.

However, optimal treatment duration for nocardiosis remains contentious. Our patient’s subsequent course vividly illustrates the critical controversy surrounding the initial intensive phase duration. Despite excellent clinical and radiological response, premature de-escalation occurred at day 18 from admission after 10 days of combination treatment when she was discharged on oral TMP–SMX monotherapy following patient insistence and lack of oral imipenem alternatives. This decision proved consequential: she returned on post-discharge day 3 with recurrent fever. Crucially, investigations excluded treatment failure due to drug resistance (*N terpenica* susceptible to imipenem [MIC ≤ 2 µg/mL] and TMP–SMX [MIC ≤ 0.5/9.5 µg/mL]) or subtherapeutic TMP–SMX levels (therapeutic sulfamethoxazole trough). The immediate relapse after carbapenem discontinuation – despite ongoing effective monotherapy – strongly implicates insufficient intensive combination therapy duration as the cause.

Literature recommendations for treatment duration show considerable variability. While prolonged total therapy (6–12 months or longer) is universally recommended for disseminated disease, particularly in immunocompromised hosts,^[18]^ the optimal duration of the initial intensive combination phase remains less defined. Guidelines typically suggest 3 to 6 weeks for severe or disseminated cases before step-down to oral monotherapy.^[19]^ Our patient’s relapse after only 10 days of combination therapy – despite profound clinical improvement, confirmed susceptibility, and therapeutic drug levels – definitively shows that rapid symptomatic and radiographic resolution does not equate to microbiological eradication in disseminated pulmonary nocardiosis. The prompt fever resolution upon re-initiating imipenem/cilastatin plus TMP–SMX, coupled with sustained remission after completing 24 days (≈3.5 weeks) of intensive therapy, strongly supports requiring ≥3 weeks of combination therapy for disseminated pulmonary nocardiosis. This duration appears essential for adequate debridement of disseminated foci, even in immunocompetent patients with structural lung disease, regardless of excellent initial response. Premature shortening of this intensive phase carries substantial relapse risk, potentially leading to further complications. This case reemphasizes that strict adherence to the minimum recommended intensive phase duration is critical for successful outcomes.

This study has several limitations inherent to its design as a single-case report. Firstly, while the temporal association strongly suggests a causal link, the conclusion that bronchoscopy directly caused the dissemination remains associative rather than proven. Secondly, as a report of a single, rare occurrence, it cannot quantify the incidence or overall risk of this complication. Finally, the precise mechanism of dissemination (e.g., aerosolization vs direct aspiration of fluid), although theorized based on clinical evidence, was not directly observed or measured.

## 4. Conclusion

This case report details an exceptionally rare presentation of pulmonary nocardiosis caused by *N terpenica* in an immunocompetent patient with underlying bronchiectasis. It provides the first clinical evidence suggesting that bronchoscopy, particularly BAL, can disseminate localized airway *Nocardia* infection, leading to acute, severe disseminated pulmonary disease. This potential complication necessitates heightened caution when performing bronchoscopy in bronchiectasis patients where nocardiosis is suspected or confirmed. Furthermore, the case emphasizes a critical therapeutic principle: for disseminated pulmonary nocardiosis, a minimum of 3 weeks of intensive combination antimicrobial therapy is essential to prevent relapse, irrespective of a rapid initial clinical response, confirmed susceptibility, and adequate drug levels. Adherence to recommended durations for the intensive phase is paramount for successful outcomes.

## Author contributions

**Data curation:** Jie Tian, Gaobing Yu.

**Funding acquisition:** Jie Tian.

**Investigation:** Jie Tian, Jingjun Dong.

**Resources:** Jie Tian, Jingjun Dong, Gaobing Yu.

**Supervision:** Wei Guan.

**Writing – original draft:** Jie Tian.

**Writing – review & editing:** Wei Guan.
